# Outbreak of coronavirus disease 2019 (COVID-19) among operating room staff of a tertiary referral center: An epidemiologic and environmental investigation

**DOI:** 10.1017/ice.2021.116

**Published:** 2021-03-19

**Authors:** April N. McDougal, Dana Elhassani, Mary Ann DeMaet, Shirley Shores, Kenneth S. Plante, Jessica A. Plante, Richard Pyles, Scott C. Weaver, Natalie Williams-Bouyer, Brenda J. Tyler, Hollie R. Davis, Janak Patel

**Affiliations:** 1 Department of Infection Control and Healthcare Epidemiology, University of Texas Medical Branch, Galveston, Texas; 2 Division of Infectious Disease, Department of Internal Medicine, University of Texas Medical Branch, Galveston, Texas; 3 World Reference Center for Emerging Viruses and Arboviruses and Department of Microbiology and Immunology, University of Texas Medical Branch, Galveston, Texas; 4 Division of Clinical Microbiology, Department of Pathology, University of Texas Medical Branch, Galveston, Texas; 5 Division of Infectious Disease, Department of Pediatrics, University of Texas Medical Branch, Galveston, Texas

## Abstract

**Objective:**

Investigate an outbreak of coronavirus disease 2019 (COVID-19) among operating room staff utilizing contact tracing, mass testing for severe acute respiratory coronavirus virus 2 (SARS-CoV-2), and environmental sampling.

**Design:**

Outbreak investigation.

**Setting:**

University-affiliated tertiary-care referral center.

**Patients:**

Operating room staff with positive SARS-CoV-2 molecular testing.

**Methods:**

Epidemiologic and environmental investigations were conducted including contact tracing, environmental surveys, and sampling and review of the operating room schedule for staff-to-staff, staff-to-patient, and patient-to-staff SARS-CoV-2 transmission.

**Results:**

In total, 24 healthcare personnel (HCP) tested positive for SARS-CoV-2, including nurses (29%), surgical technologists (25%), and surgical residents (16%). Moreover, 19 HCP (79%) reported having used a communal area, most commonly break rooms (75%). Overall, 20 HCP (83%) reported symptomatic disease. In total, 72 environmental samples were collected from communal areas for SARS-CoV-2 genomic testing; none was positive. Furthermore, 236 surgical cases were reviewed for transmission: 213 (90%) had negative preoperative SARS-CoV-2 testing, 21 (9%) had a positive test on or before the date of surgery, and 2 (<1%) did not have a preoperative test performed. In addition, 40 patients underwent postoperative testing (mean, 13 days to postoperative testing), and 2 returned positive results. Neither of these 2 cases was linked to our outbreak.

**Conclusions:**

Complacency in infection control practices among staff during peak community transmission of SARS-CoV-2 is believed to have driven staff-to-staff transmission. Prompt identification of the outbreak led to rapid interventions, ultimately allowing for uninterrupted surgical service.

Identified in China’s Hubei province, severe acute respiratory syndrome coronavirus 2 (SARS-CoV-2) is the RNA β-coronavirus virus that causes coronavirus disease-19 (COVID-19).^1^ Data principally support transmission via respiratory droplets and aerosols, with a median incubation period of 5 days (range, 2–12 days).^2–4^

Our healthcare facility is a 623-bed, academic, tertiary-care center composed of 61 departments and 20 surgical suites. All employees are required to wear hospital-provided face masks, to maintain social distancing, and to perform daily symptom-free attestations upon workplace entry. Unless eating or drinking, when social distancing should be maintained, face masks are required for all interpersonal interactions. It is well known that healthcare personnel (HCP) are at increased risk for acquiring SARS-CoV-2 in the United States^5^; 55% of HCP with COVID-19 report only work-related exposures.^6^ Thus, it is imperative that HCP follow institutional infection control policies to reduce the risk of acquisition.

On August 13, 3 symptomatic operating room (OR) HCP were found to be positive for SARS-CoV-2. Following identification of this cluster, the Department of Infection Control and Healthcare Epidemiology (ICHE) led an investigation that included contact tracing, mass employee testing, and environmental sampling. We hypothesized that the outbreak was due to horizonal transmission among HCP secondary to decreased compliance with infection control practices in shared communal areas. Additionally, we aimed to determine whether recent institutional changes to the SARS-CoV-2 preoperative patient testing algorithm increased risk of patient-to-staff transmission and, because of the burden of positive HCP, staff-to-patient transmission. Institutional review board approval was not needed for this study. Herein, we describe our outbreak investigation.

## Methods

### Epidemiological investigation

The cluster of 3 HCP positive for SARS-CoV-2 was identified via a generated daily report. A cluster is defined as at least 2 cases occurring in the same location or department within a 2-week period. The daily report compiles all positive SARS-CoV-2 molecular tests performed in the prior 24 hours at our institution, including all satellite locations, and contains a preliminary count of affected HCP. The report is shared with Employee Health and the Office of Institutional Compliance, where employment status is verified. An ICHE staff member contacts the HCP via telephone for contact tracing and risk assessment. At the time of this investigation, high-risk exposure was defined as exposure to a known positive SARS-CoV-2 case in which both the exposed and infected individuals, whether symptomatic or asymptomatic, were unmasked and within ˜2 m (6 feet) of one another for >15 minutes.^7^ Inquiry included any potential household, community, and known hospital exposures (patients or staff) as well as exposure to hospital break rooms, locker rooms, and the physician work room. Additionally, HCP were asked about nonoccupational high-risk activities including attending large gatherings or dining in restaurants.

Employee mass testing began on day 3 of the outbreak and continued through the day 5. Voluntary testing was available to all hospital employees, not just OR staff. Nasopharyngeal swabs were collected and tested using a SARS-CoV-2 isothermal nucleic acid amplification assay.

**Fig. 1.**
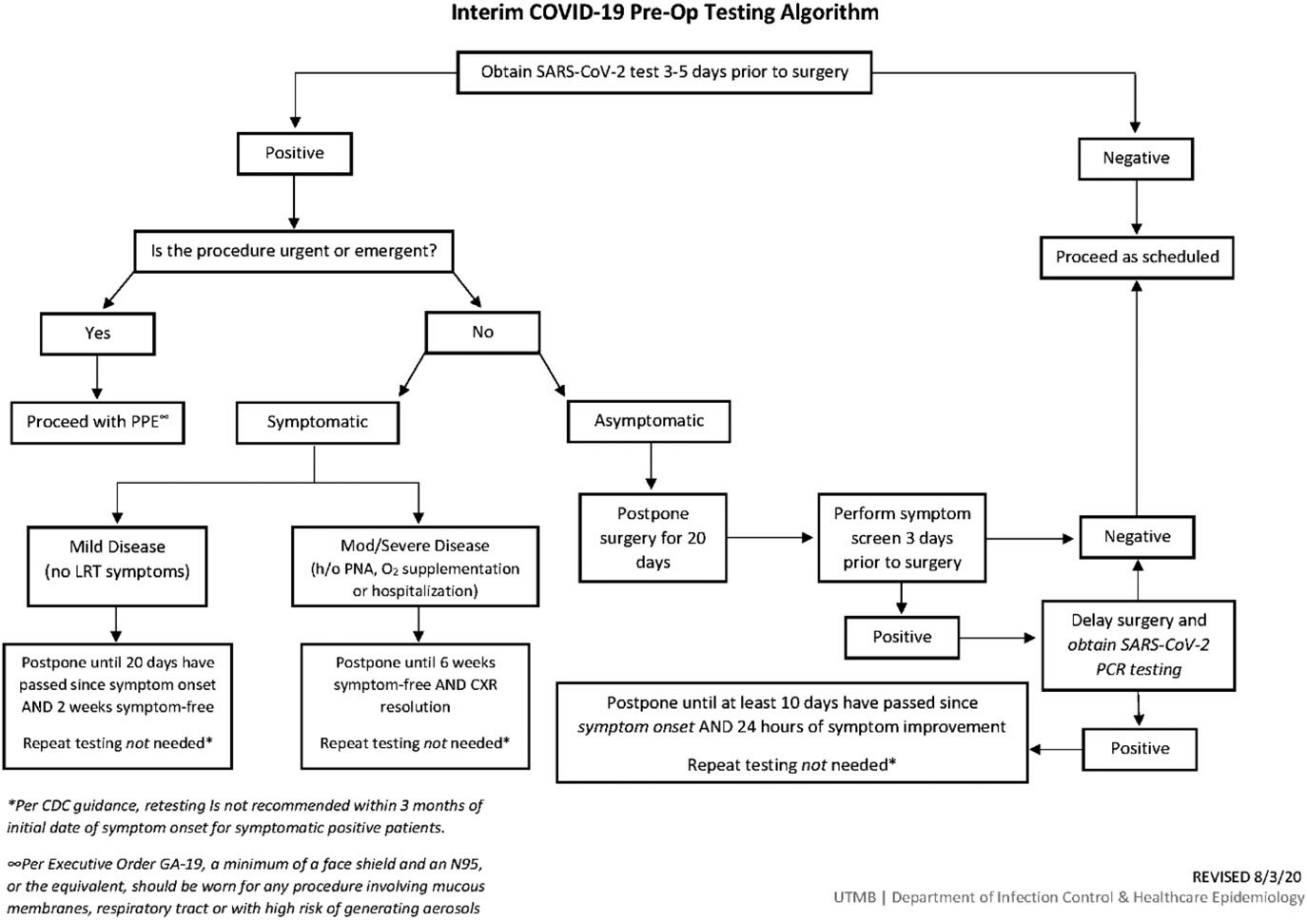
Preoperative SARS-CoV-2 testing algorithm for surgical cases.

Furthermore, because all SARS-CoV-2–positive HCP worked in the OR and because there had been recent changes to the patient preoperative SARS-CoV-2 testing algorithm (Fig. 1), we sought to investigate potential staff-to-patient and patient-to-staff transmission. We reviewed all surgical cases performed during the 7 days preceding the cluster of positive HCP were reviewed for date of surgery, surgery type, surgical service, surgical team including all staff present for the operation, operating room number, preoperative SARS-CoV-2 status including date of positive testing and postoperative SARS-CoV-2 status (if obtained). Because most individuals have detectable virus within 7 days of exposure to SARS-CoV-2, we reviewed the cases of patients who had surgery during this period.^1^

### Environmental investigation

Surveys and environmental samples were collected from the OR staff and physician break rooms, staff and physician locker rooms (men and women) and the physician work room. All locations are in the same area of the hospital, 1 floor above the operating rooms, and they are occupied by HCP between surgical cases and shifts (Supplementary Fig. S1 online).

### Environmental virological analysis

Environmental sampling was performed using Copan 480 C Eswabs (lot 192073000, batch 171P50). In most instances, an item or handle was swabbed. In the case of flat surfaces (table or door push plate), a 5-cm square was swabbed. The only exception were 2 benches in the women’s staff locker room, which were swabbed up and down their full length. After sampling, the swab was deposited into a 15 mL conical container with 2 mL media composed of Dulbecco’s minimal essential medium (DMEM) with 2% fetal bovine serum (FBS) and 1% antibiotic-antimycotic (VTM). Swabs were held in a portable chest maintained at 4°C during the collection period (˜3 hours).

Immediately following sample completion, the freshly collected environmental samples were sent to the processing laboratory. Within a biosafety cabinet, the samples were mixed in a vortexer for 3 seconds before 50-µL aliquots were transferred to individual wells of a skirted, 96-well, PCR plate (Thermofisher Scientific, Waltham, MA) containing 50 µL nuclease-free water creating a total volume of 100 µL. The plate was sealed with foil tape and transferred to a C1000 thermocycler (Bio-Rad, Hercules, CA) and heated for 15 minutes at 95°C. The processed sample plate then was briefly centrifuged and used as template for RT-PCR amplification. A positive control (VTM spiked with 1E5 genomes of inactivated WA-1 SARS-CoV-2) and a negative control (50 µl sterile VTM) were included for the run.

### Reverse-transcription, real-time polymerase chain reaction (RT-PCR)

A 10-µL aliquot of prepared template was added to a master mix solution containing 5.0 µL of 4x Reliance One-Step Multiplex Supermix (Bio-Rad), 1.0 µL forward and reverse primers (5.0 µM), 1.0 µL 7.5 µM FAM-labeled Taqman Probe, and 2.0 µL nuclease-free water to a final volume of 20 µL (Supplementary Table S1 online) within a 96-well qPCR plate (Bio-Rad). RT-PCR standards, serving as positive controls, were included for each target and consisted of synthetic RNA covering the regions of interest in the SARS-CoV-2 genome. NP-tested negative clinical material was used as a host extraction and PCR control. Negative controls for the PCR reaction (NTC) were also included. Each prepared plate (1 per target) was sealed with optical tape then pulse centrifuged and placed in a CFX96 real-time instrument (Bio-Rad). After an initial reaction at 50°C for 10 minutes to complete reverse transcription, the samples were subjected to a cycle of 95°C for 10 minutes followed by 45 replicate cycles consisting of 2 steps programmed for 10 seconds at 95°C, then 30 seconds at 60°C. A plate-read step was included after each 60°C cycle. The limit of detection for this approach was 10 genomes per reaction or 1,000 genomes/mL starting material.

## Results

### Epidemiological investigation

**Fig. 2.**
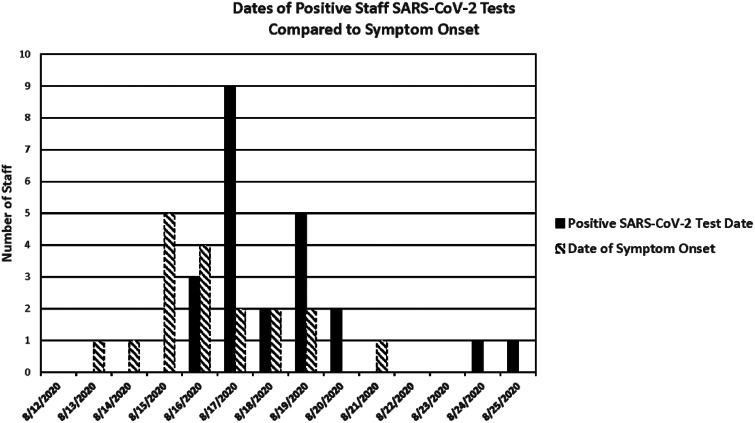
Epidemic curve demonstrating positive SARS-CoV-2 test dates with symptom onset dates among positive staff.

Over 9 days, 24 HCP tested positive for SARS-CoV-2. Table 1 summarizes each case’s characteristics and epidemiologic history. Men (54%) were slightly more affected than women (46%). The most frequently implicated roles were nurses (29%) followed by surgical technologists (25%) and surgical residents (16%). Among these 24 HCP, 5 (20%) recalled exposure to a known positive contact. Also, 19 HCP (79%) reported using one of the communal areas investigated, most commonly break rooms (75%), and 3 (12.5%) reported other high-risk nonoccupational exposures such as large gatherings or dining in a restaurant. Furthermore, 13 HCP (54%) reported unmasked, non–socially distanced interactions with other HCP in at least 1 of the communal areas investigated. Overall, 20 HCP (83%) reported symptoms. Figure 2 compares HCP symptom onset day to the day of positive SARS-CoV-2 test.

**Table 1.** Line List of Identified SARS-CoV-2 Positive Staff Including Professional Position, Date of Positive SARS-CoV-2 Testing, and Symptom Onset and Exposure History

Employee (n = 24)	Sex	Professional Title	Date of Positive SARS-CoV-2 Test	Identify During Mass Testing	Date of Symptom Onset	Exposure to Known Positive Contacts	Exposure to Large Gatherings or Hospital Common Locations	Confirmed Unmasked Exposure and Social Distancing Not Maintained
1	Male	Physician assistant	8/16/2020	No	8/15/2020	No	Unknown	No
2	Female	Nurse	8/16/2020	No	8/14/2020	No	Break room	Yes
3	Male	Surgical technologist	8/16/2020	No	8/15/2020	No	Break room	Yes
4	Male	Resident	8/17/2020	No	8/15/2020	Patient Employee	Break room	Yes
5	Female	Surgical technologist	8/17/2020	No	8/13/2020	No	Break room	Yes
6	Female	Patient care technician	8/17/2020	No	8/15/2020	No	Break room	Yes
7	Female	Nurse	8/17/2020	No	8/16/2020	No	Break room	Yes
8	Male	Nurse	8/17/2020	No	8/16/2020	No	Break room	Yes
9	Male	Coordinator	8/17/2020	No	8/16/2020	No	Break room Large gathering	Yes
10	Male	Nurse	8/17/2020	No	8/15/2020	No	Break room	No
11	Male	Patient care technician	8/17/2020	No	Unknown	Unknown	Unknown^a^	Unknown
12	Male	Pharmacist	8/17/2020	No	8/16/2020	No	Break room	Yes
13	Female	Nurse	8/18/2020	No	8/17/2020	No	Breakroom	Yes
14	Female	Surgical technologist	8/18/2020	No	8/17/2020	No	Break room Large gathering	Yes
15	Female	Resident	8/19/2020	No	8/18/2020	Patient	Break room	No
16	Female	Nurse	8/19/2020	Yes	8/18/2020	No	Break room Restaurant	No
17	Male	Nurse	8/19/2020	Yes	8/18/2020	No	Break room	Yes
18	Male	Surgical technologist	7/2/2020^b^; 8/19/2020	Yes	6/2020	No	Break room	Yes
19	Female	Resident	8/19/2020	No	8/19/2020	Employee	Unknown	No
20	Male	Faculty	8/19/2020	Yes	8/19/2020	Spouse	No	No
21	Female	Resident	8/20/2020	Yes	Asymptomatic	Patient	No	No
22	Male	Medical student	8/20/20/20	Yes	Asymptomatic	No	Computer room	No
23	Male	Surgical technologist	8/25/2020	No	8/17/2020	No	Break room	No
24	Female	Surgical technologist	8/24/2020	No	8/21/2020	No	Break room	No

a Individual contacted without return of call, unable to assess.

b Individual originally tested positive on 7/2/2020 and was retested as part of the investigation. Symptom onset began in late June 2020.

More than half of the cases were identified prior to mass testing. In total, 215 hospital employees underwent testing (Supplementary Table S2 online) and 6 (3%) were positive; 5 were OR HCP and 1 was a medical student (Table 1). In total, 78 OR support staff were scheduled to work during the mass testing, and 39 (50%) underwent testing. Of the 215 HCP who were tested, 82 (38%) were surgical service physicians, residents, and medical students. Also, 6 HCP were identified outside mass testing. One HCP had originally been diagnosed with COVID-19 on July 2. They elected to undergo repeat testing as part of this investigation. Although it was included in this investigation, this positive result was not believed to have been a reinfection. Another HCP could not be reached for interview. The last positive HCP related to this outbreak was identified 9 days after the index cluster.

In total, 236 patients underwent surgery in our ORs in the 7 days before the index cluster. Of these, 213 cases (90%) had a negative SARS-CoV-2 test, 21 (9%) had a positive test before or on the date of surgery, and 2 (<1%) did not have a test in the system. Table 2 includes those who had a positive test any time before or on the date of surgery. Of these, 15 (71%) were positive >20 days from time of surgery and 6 (29%) tested positive within 20 days of the date of surgery. Days of positivity until surgery ranged from 0 to 105, with a median of 31 days and a mean of 35 days. In addition, 9 cases (41%) had at least 1 positive employee assisting with their surgery. Of these, 8 positive results were in patients who had tested positive >20 days from time of surgery, and 1 was in a patient who tested positive within 20 days of surgery. Of the cases testing positive within 20 days of surgery, only 1 surgery was performed outside OR 13, the designated SARS-CoV-2 suite, where full personal protective equipment, including N95 respirators, must be worn. No HCP involved in this case tested positive for SARS-CoV-2.

**Table 2.** Surgical Patients With Positive SARS-CoV-2 Testing Before or at the Time of Surgery

Surgical Case No. (n=21)	Date of Positive SARS-CoV-2 Test	Date of Surgery	Total Days of Positivity at Time of Surgery	Operating Room Used	No. of Positive Surgical Staff Involved with Surgical Case
1	8/9/2020	8/9/2020	0	13	0
2	8/8/2020	8/9/2020	1	17	0
3	6/23/2020	8/10/2020	48	20	0
4	8/9/2020	8/10/2020	1	13	0
5	6/22/2020	8/11/2020	49	18	0
6	6/7/2020	8/11/2020	65	14	1
7	7/11/2020	8/11/2020	31	18	0
8	6/23/2020	8/12/2020	50	16	1
9	8/2/2020	8/12/2020	10	13	0
10	6/23/2020	8/12/2020	50	1	1
11	5/26/2020	8/12/2020	78	1	1
12	8/12/2020	8/13/2020	1	13	2
13	7/23/2020	8/13/2020	21	18	1
14	8/3/2020	8/14/2020	11	13	0
15	7/8/2020	8/14/2020	37	10	1
16	6/23/2020	8/14/2020	52	15	0
17	7/23/2020	8/14/2020	22	1	0
18	5/1/2020	8/14/2020	105	1	0
19	7/23/2020	8/14/2020	22	1	0
20	6/25/2020	8/14/2020	50	6	2
21	7/21/2020	8/14/2020	24	1	1

Of the 213 preoperative negative SARS-CoV-2 cases, 36 (17%) obtained postoperative testing, 2 of whom were positive. As noted in Table 3, no HCP involved in the first case tested positive. The second case included 4 positive HCP. Of the 21 cases with a positive test prior to surgery, 4 had repeat testing performed postoperatively, of whom 1 converted negative.

**Table 3.** Surgical Patients Who Tested Positive for SARS-CoV-2 Postoperatively, the Surgical Operating Room Used and Number of Identified Positive Staff Assisting in the Surgical Case Prior to Staff Testing Positive

Surgical Case No. (n = 2)	Date of Negative Preoperative SARS-CoV-2 Test	Date of Surgery	Date of Positive Postoperative SARS-CoV-2 Test	Total days from Surgery to Positivity	Operating Room Used	No. of Positive Surgical Staff Involved with Surgical Case
1	8/10/2020	8/12/2020	8/19/2020	7	10	0
2	8/11/2020	8/14/2020	8/16/2020	2	17	4

### Environmental investigation

In total, 72 environmental samples were collected and tested. CFX Maestro version 1.1 software (Bio-Rad) was used to collect data and establish the cycle threshold (Ct) based on the placement of a baseline. Values <42 Ct indicated a positive amplification detection. None of the samples was positive for SARS-CoV-2 genomic material.

## Discussion

Our investigation details potential modes of SARS-CoV-2 transmission among 24 OR HCP at an academic medical center. The importance of maintaining masking and social distancing among HCP is imperative despite periods of declining community incidence. Galveston County’s SARS-CoV-2 test positivity peaked the week of June 28–July 4 at 12%. At the time of this outbreak, the county’s test positivity rate was 6%.^8^ SARS-CoV-2 community incidence and hospitalizations have declined at the time of this writing.

The environmental investigation included the OR staff and physician break rooms, staff and physician locker rooms (men’s and women’s), and the physician work room. These locations were targeted for staff-to-staff transmission given prior cluster investigations at our institution. Internally, smaller outbreaks have been linked to communal areas like break rooms because these shared spaces facilitate unmasked person-to-person interaction during meals. Literature on fomite transmission and environmental contamination in SARS-CoV-2 propagation is inconclusive but suggests an unlikely major role.^4,9^ Our surveys were remarkable for complacency in infection control practices among HCP including unmasking when not eating or drinking, not maintaining social distancing in the identified common areas, and presenting to work symptomatic. We believe HCP relaxed their practices due to the declining SARS-CoV-2 community incidence and because of occupational fatigue surrounding COVID-19. Our institution abides by the Families First Coronavirus Response Act (FFCRA), which provides employees fully paid sick leave while quarantined.^10^ However, with new residents eager to start training, dismissal of minor symptoms like nasal congestion and headache that could be attributed to other conditions (eg, seasonal allergies) had occurred. Potential stigma associated with missing work could have also contributed to staff coming to work symptomatic.

Despite the negative environmental samples for SARS-CoV-2 genomic material, we strongly believe that most transmission occurred in communal areas. This outbreak required immediate intervention to halt transmission. Environmental services (EVS) staff thoroughly cleaned the communal areas on day 2 of the investigation and increased area cleaning to 4 times daily from once or twice daily thereafter. Antimicrobial wipes were made available to encourage staff to clean high-touch surfaces between use. Prior to this, no cleaning agents were available in these areas. The environmental samples tested were collected 3 days after implementing these enhanced cleaning methods. The areas had been cleaned roughly 10 times before sampling occurred. Additionally, 22 positive HCP had been identified and relieved from work by the time of sampling. Knowing these limitations, we planned to perform air sampling.^9,11^ Equipment was ordered however due to the mandatory evacuation order for Hurricane Laura the day sampling was to occur, it was not performed.

A high percentage of symptomatic staff was identified. This finding is important because HCP frequently reported symptom onset during times they were scheduled to be working (Fig. 2). In lieu of in-person screeners, employees self-attest daily they are symptom-free using either a kiosk or the UTMB app and provided a face mask daily when presenting to work. Symptom screening includes cough, shortness of breath, fever, chills, muscle pain, headache, sore throat, and loss of taste or smell. If staff have symptoms consistent with COVID-19 they are instructed to stay home or are released from duty if symptoms develop at work, and they are instructed to contact their supervisor and Employee Health for testing. Given the high rate of symptomatic HCP, one could extrapolate the “true” cohort being much larger since asymptomatic infection has been estimated at 30% to 45%. Additionally, asymptomatic individuals may have prolonged viral shedding.^9,12^ Using this information, the infected cohort may have included as many as 40 HCP. An outbreak of this size would likely have secondary impacts on community spread. Thankfully, no HCP deaths or hospitalizations resulted from this outbreak.

Mass testing rapidly identified 25% of our positive HCP. Had there been further delay in case detection, additional transmission would have resulted in closing the OR, which would have severe impacts on patient care in this regional referral center. This situation is not unprecedented during the COVID pandemic.^13^ Timely identification and implementation of control measures were key to limiting transmission and thus allowed uninterrupted operations.

Ultimately, we did not find evidence that changes to our patient preoperative SARS-CoV-2 testing algorithm were related this outbreak. The update included testing all preoperative patients with a PCR-based test 3–5 days before surgery unless previously positive in the preceding 3 months. Unless urgent or emergent, all new positive patients’ surgeries are delayed a minimum of 20 days. Asymptomatic positives are rescreened for symptoms prior to their rescheduled surgery. If symptoms are present, testing is repeated, and if the result is positive, surgery is delayed 10 additional days.

Of the 21 patients with a SARS-CoV-2 positive test prior to surgery, 9 had at least 1 positive employee assisting in their case. Nearly half of HCP developed symptoms before August 16, suggesting an exposure time around August 11. Among the preoperative SARS-CoV-2–positive surgical cases operated on prior to this date, 3 were in individuals diagnosed <20 days before time of surgery. These patients would have had higher viral shedding than those operated on >20 days since diagnosis.^12,14^ Of these 3 cases, 2 underwent surgery in the designated COVID-19 OR. No positive HCP were identified related to these 3 cases. Of the 8 surgical patients testing positive >20 days before surgery, 4 surgeries were performed before August 11. Only 1 case involved a positive HCP. Despite the algorithm, this patient underwent repeat testing the day of his surgery and SARS-CoV-2 was not detected.

Several systematic advantages led to rapid containment of this outbreak, primarily the institutional transparency and multidepartment collaboration among the following departments: Department of Surgery, ICHE, Employee Health, World Reference Center for Emerging Viruses and Arboviruses, Galveston National Laboratory, Department of Microbiology, and Environmental Services. These departments facilitated the prompt identification outbreak as well as interventions resulting in a total of 9 days from detection of the index cluster to when the last case was identified. The proximity and history of collaboration with the Galveston National Laboratory provided access to a unique set of environmental testing resources that are not available to most healthcare systems. This partnership truly sets our investigation apart from others.

Our investigation had several limitations. Environmental cleaning was performed prior to environmental sampling. Furthermore, the mandatory evacuation order for Hurricane Laura prevented air sampling. Although the mass testing event was well attended, its voluntary nature resulted in <50% participation by the target population (per staffing roster), suggesting other possible undetected cases among staff. At the time of this investigation, we could not mandate employee testing. We have since garnered institutional support for mandated screening during workplace outbreaks. Additionally, those interviewed reported few exposures to known positive contacts at or outside work. This incidence may be falsely low given possible reluctance to admit participation in high-risk activities and/or contact with known positive individuals. Another limitation is the passive surveillance used postoperatively in the surgical patients. Postoperative testing was dependent upon patients independently seeking testing or requiring testing per policy for their care.

We were able to mitigate this outbreak because of prompt identification of the index cluster, mass testing and swift interventions including reeducation about masking, maintaining social distancing, limiting capacity in communal areas, remaining off duty when feeling ill, and increased environmental cleaning. No additional cases have been identified since implementing these measures.

Our investigation revealed that HCP noncompliant with infection control practices in shared communal areas and presenting to work symptomatic led to staff-to-staff transmission of SARS-CoV-2. Evidence of patient-to-staff transmission causing the outbreak due to recent changes to the institution’s preoperative SARS-CoV-2 testing algorithm could not be found. Furthermore, no staff-to-patient transmission was identified in our investigation. Our investigation highlights the importance of maintaining infection control measures despite declining SARS-CoV-2 incidence for the safety of both HCP and patients.

## Supplementary material

For supplementary material accompanying this paper visit http://dx.doi.org/10.1017/ice.2021.116. click here to view supplementary material
