# Supplementary material for: Outbreak of coronavirus disease 2019 (COVID-19) among operating room staff of a tertiary referral center: An epidemiologic and environmental investigation
Source: Infect Control Hosp Epidemiol. 2021 Mar 19:1–7. doi: 10.1017/ice.2021.116 (PMC8042383; doi:10.1017/ice.2021.116)
Supplement: Supplementary file 1 [file S0899823X21001161sup001.docx]

##### SUPPLEMENTAL MATERIAL (Online-Only Material)

##### Supplementary Fig. S1. Schematic diagram of areas used by the operating room staff between surgical cases and shifts. Starred are staff lounges, locker rooms and the physician work room.

**Supplementary Table S1.** SARS-CoV-2 Probes are Labeled at the 5´-End With the Fluorophore 9-Carboxyfluroescein (6-FAM). An internal quencher (ZEN) and a 3´-end quencher (IowaBlackFQ, IABkFQ)^a^

| COVID-19  SARS-CoV-2 Primer | Description | Gene  Target | Oligonucleotide Sequence (5´>3´) | Label^a^ | Working  Concentration | Final Concentration  (20 µL) | Origin |
| --- | --- | --- | --- | --- | --- | --- | --- |
| 2019-nCoV_N2-F | FP | N2 | TTA CAA ACA TTG GCC GCA AA | None | 5 µM | 0.25 µM | CDC |
| 2019-nCoV_N2-R | RP | N2 | GCG CGA CAT TCC GAA GAA | None | 5 µM | 0.25 µM | CDC |
| 2019-nCoV_N2-P | Probe | N2 | 6-FAM/ACA ATT TGC /ZEN/CCC CAG CGC TTC AG/IABkFQ | 6-FAM, ZEN, IABkFQ | 7.5 µM | 0.375 µM | CDC |
| E_Sarbeco_F1 | FP | E | ACA GGT ACG TTA ATA GTT AAT AGC GT | None | 5 µM | 0.25 µM | WHO |
| E_Sarbeco_R2 | RP | E | ATA TTG CAG CAG TAC GCA CAC A | None | 5 µM | 0.25 µM | WHO |
| E_Sarbeco_P | Probe | E | 6-FAM/ACA CTA GCC /ZEN/ATC CTT ACT GCG CTT CG/IABkFQ | 6-FAM, ZEN, IABkFQ | 7.5 µM | 0.375 µM | WHO |
| orf-8-F | FP | orf8 | AAT CAG CAC CTT TAA TTG AAT TG | None | 5 µM | 0.25 µM | UTMB |
| orf-8-R | RP | orf8 | CAG GAA ACT GTA TAA TTA CCG ATA | None | 5 µM | 0.25 µM | UTMB |
| orf-8-P | Probe | orf8 | 6-FAM/TGA GGC TGG /ZEN/TTC TAA ATC ACC/IABkFQ | 6-FAM, ZEN, IABkFQ | 7.5 µM | 0.375 µM | UTMB |
| RNAse P-F | FP | RNAse P | AGA TTT GGA CCT GCG AGC G | None | 5 µM | 0.25 µM | CDC |
| RNase P-R | RP | RNAse P | GAG CGG CTG TCT CCA CAA GT | None | 5 µM | 0.25 µM | CDC |

Note. FP, forward primer; RP, reverse primer; CDC, Centers for Disease Control and Prevention; WHO, World Health Organization; UTMB, University of Texas Medical Branch.

^a^All primes and probes were purchased from Integrated DNA Technologies (Coralville, IA).

**Table S2.** Number of Employees Tested at the Mass Testing Event by Professional Title and Department

| Professional Title | Sample Size, No. (%) |
| --- | --- |
| Physician | 86 (40) |
| Nurse | 49 (23) |
| Other^a^ | 19 (9) |
| Sterile processing technician | 16 (7) |
| Surgical technologist | 14 (7) |
| Patient care technician^b^ | 12 (6) |
| Medical student | 8 (3) |
| Administration | 7 (3) |
| Anesthesia technologist | 4 (2) |
| Total | 215 (100) |
| Department | Sample Size No. (%) |
| OR-related staff | 190 (88) |
| Non–OR-related staff | 25 (12) |

Note. OR, operating room.

^a^Materials management, environmental services (EVS), and perfusionists.

^b^Patient care technician also includes emergency department technicians and medical assistants.
